# Legumes—A Comprehensive Exploration of Global Food-Based Dietary Guidelines and Consumption

**DOI:** 10.3390/nu14153080

**Published:** 2022-07-27

**Authors:** Jaimee Hughes, Emma Pearson, Sara Grafenauer

**Affiliations:** 1 Grains & Legumes Nutrition Council, 1 Rivett Rd., North Ryde 2113, Australia; 2 School of Medical, Indigenous and Health Sciences, University of Wollongong, Northfields Avenue, Wollongong 2522, Australia; emma.pearson01@gmail.com; 3 School of Health Science, Faculty of Medicine and Health, University of New South Wales, Randwick 2052, Australia; s.grafenauer@unsw.edu.au

**Keywords:** legumes, dietary guidelines, consumption, sustainability, chronic disease

## Abstract

Despite the well-known human and planetary health benefits of legumes, consumption is often low. This scoping review aimed to evaluate the inclusion of legumes in global food-based dietary guidelines (FBDG), and to review consumption data against global food group classifications for legumes. The review of FBDG from 94 countries identified legume-based key messaging, the key terms used to define legumes, recommended serving size and frequency of consumption and the classification of legumes into food groups as depicted by food guides. The 2018 Global Dietary Database isolated consumption data of legumes and beans using individual-level, nationally representative dietary survey data for matched countries. Food-based dietary guidelines from 40/94 countries most often identified legumes utilising the term legumes, followed by beans (*n* = 13), pulses (*n* = 10), or as beans, peas and lentils (*n* = 5). The serving size recommendations for legume consumption varied widely, and there was no consistency in the suggested frequency of consumption. Median bean and legume consumption for countries with FBDG ranged from 1.2 g/d (Norway) to 122.7 g/d (Afghanistan). Classification of legumes into food groups varied, with 38% of countries categorising legumes in the protein-rich food group, 20% were in a group on their own and 15% were in the starchy staples group. In countries where legumes were together with either nuts or seeds had the greatest range in intake (11.6–122.7 g/day), followed by those that grouped legumes together with protein-rich foods (4.0–104.7 g/day), while countries that grouped legumes into two food groups, in an attempt to promote consumption, tended to have a lower consumption. Greater emphasis and perhaps repositioning of legumes in dietary guidelines may be required to encourage consumption for health, environmental and economic benefits.

## 1. Introduction

Legume, from the Fabacae (or Leguminosae) botanical family, is the more inclusive classification for non-oil seed (pulse) and oil-seed crops (peanuts and soybeans) and includes both fresh and dried forms [1]. Common types of legumes include dried beans, broad beans, peas, chickpeas, cowpeas, lentils, lupins, peanuts and soybeans. Abundant in various vitamins and minerals, including B vitamins, iron, magnesium, potassium and zinc, legumes are an economical source of dietary fibre, phytonutrients and protein important for plant-based or flexitarian dietary patterns [2,3]. According to the Food and Agriculture Organization of the United Nations (“FAO”), legumes are an important inclusion in the diet with known benefits for human and planetary health [3]. Regular consumption has been shown to improve the nutrient density of the diet in a US population [4], and has been linked to reductions in the risk of disease, particularly coronary heart disease [5] and all-cause mortality at a serving of 50 g per day [6]. Soy intake specifically is linked to a reduced risk of certain cancers including prostate and breast cancer [7], where population studies indicate soy consumption has a role in both preventing breast cancer and reducing risk of reoccurrence in breast cancer survivors due to the isoflavone content [8,9]. Legumes also play a valuable role in sustainable food production, and are well placed to form part of nutritious and environmentally sustainable dietary patterns [10,11], as highlighted in the recent Eat Lancet Commission Planetary Health Diet, where it is recommended to consume 100 g/day (50 g of dried beans, lentils and peas, 25 g of soybeans and 25 g of peanuts) [12]. Increased intakes have also been shown to have a significant impact on socioeconomic burden [13], with a recent Canadian study reporting potential combined annual health care and lost productivity cost savings of CAD377.9 million in the prevention of type 2 diabetes and cardiovascular disease with the daily consumption of 100 g of legumes [13]. Lower targets, of 50 g per day based only on Coronary Heart Disease (CHD) in an Australian population have also been shown to produce cost savings of AUD 4.3 (95% CI 1.2–7.4) to AUD 85.5 (95% CI 23.3–147.7) million annually [14].

National food-based dietary guidelines (“FBDG”) provide advice on foods, food groups, and dietary patterns for populations to promote overall health [15]. Although included in earlier dietary guidance as part of the Five Food Groups, legumes were first mentioned in the 1992 Australian Dietary Guidelines (“ADG”) as part of the vegetable food group (“Eat plenty of breads and cereals (preferably whole grain), vegetables (including legumes), and fruits”) [16]. In the most recent revision of the ADG in 2013, legumes were placed into two food groups; as both a vegetable (75 g serving size) and as an alternative to meat within the protein-rich food group (150 g serving size), with the aim of encouraging consumption [17]. Despite this, modelling as part of the development of the ADGs suggests that Australian’s would need to increase consumption by 470% in order to meet the proposed recommendations [18]. Although there is no universally accepted daily target, a recent review conducted by Marinangeli et al. (2016) concluded that a reasonable serve size to be promoted internationally could be based on 100 g (1/2 metric cup or 125 mL) of cooked beans, lentils, chickpeas, or peas per day [19]. This serve size guide was deemed appropriate based on the nutrient content, particularly the delivery of iron, folate and zinc, and is sufficient to facilitate nutrient content claims across multiple jurisdictions [19].

While the classification of legumes as both a vegetable and a protein source in the ADGs was intended to encourage consumption, such placement may be confusing for consumers and nutrition professionals due to their distinct nutritional profile [11]. It has been suggested that future recommendations to encourage legume intake into dietary patterns may require more explicit emphasis in FBDG [20]. Therefore, this scoping review aimed to evaluate the inclusion of legumes in FBDG globally, and to review country- and regional-level consumption data against global food group classifications for legumes.

## 2. Materials and Methods

### 2.1. Legume Messaging in Food-Based Dietary Guidelines

To review legume-based messaging within National Dietary Guidelines, the FAO online repository of FBDG was utilised to source data for countries with dietary recommendations [15]. Only those related to the general healthy population were included for review. In July and August 2021, country-specific webpages from the FAO online repository were individually reviewed, and relevant data were extracted and transcribed into a Microsoft^®^ Excel^®^ spreadsheet (Microsoft 365 MSO Version 16.0.13426.20306, Redmond, WA, USA) for analysis. The following data were extracted for each country: Region, as defined by the FAO (Asia and the Pacific “APAC”, Latin America and the Caribbean “LAC”, Europe, Africa, Near East and North America), the official name of the dietary guidelines, date of most recent revision, verbatim legume-based key messages, the specific terminology used to define legumes, the recommended dietary intake of legumes, serving size suggestions and the classification of legumes into food group/s using the provided food guides. Food guides are a visual representation of the dietary guidelines and depict the recommended food groups, and often frequency of consumption for a nutritious diet [15]. Verbatim key messages related to legumes were further classified as either qualitative or quantitative (e.g., recommended frequency of consumption) based messaging. Where available, full FBDG documents in English language were consulted to obtain additional information such as recommended servings sizes and/or frequency of consumption.

The pictorial food guides for each country were visually examined to determine the classification of legumes into one of six food group/s: legumes (own group), fruits/vegetables, vegetables only, starchy staples (together with grain, cereals, tubers and/or cassava), protein-rich foods (together with lean meats, poultry, fish, eggs, tofu and/or nuts and seeds), legumes, nuts and seeds (own group), or a combination of the above mentioned. The precise terminology used to define legumes in the guidelines was also noted.

Using Microsoft^®^ Excel^®^ (Microsoft 365 MSO Version 16.0.13426.20306, Redmond, WA, USA), description statistics were applied to explore how legumes were classified and encouraged within dietary guidelines.

### 2.2. Legume Consumption and Comparison to Legume Food Group Classifications

Global bean and legume consumption data were extracted from the 2018 Global Dietary Database (“GDD 2018”) in June 2022 [21]. The GDD 2018 prediction model provides estimate consumption data for 54 dietary factors, including bean and legume data for 185 countries stratified by age, sex, country, region and time period [22]. Details on data collection for the GDD have been described in detail elsewhere [23,24,25,26]; however briefly, estimates are derived from a combination of sources including the use of ~1500 national and subnational surveys of individual-level dietary intakes from public and private sources and from over 800 *covariates* to supplement individual level dietary intake data including food and nutrient availability data and food product sales [27]. The first iteration, GDD 2010, was used to establish the calculations for the 2010 and 2013 Global Burden of Disease Study [28]. According to the GDD 2018, bean and legume consumption is defined as the total intake of beans and legumes (beans, lentils), including fresh, frozen, cooked, canned or dried beans/legumes and includes soybeans but excludes soy milk and soy protein. This definition also excludes peanuts and peanut butter [29].

Median legume intake data (in grams/day) and 95% confidence interval was extracted and analysed in Microsoft^®^ Excel^®^ (Microsoft 365 MSO Version 16.0.13426.20306, Redmond, WA, USA). Intake data for 185 countries were available; however, only those with FBDG were used in the present analysis (*n* = 94). Following extraction of data, countries were recategorised according to the FAO regional categorisation scheme as previously defined (APAC, LAC, Europe, Africa, Near East and North America) to determine regional-level findings. Intake data were analysed for individuals aged 20 years and over (both sexes), from all education levels (low (0–6 years formal), medium (6.01–12 years) and high (12.01+ years) and all residences (rural and urban).

Country and regional-level consumption data were compared to a daily target of 50 g of cooked legumes, as this target was most commonly utilised in the literature [5,6,12].

## 3. Results

A total of 94 countries with FBDG were included for review to explore how legumes were classified and encouraged within dietary guidelines (excludes Cambodia as the FBDG only related to school children aged 6 to 17 years). Of the 94 included countries, 35% were in Europe (*n* = 33), 31% were in LAC (*n* = 29), 18% were from the APAC region (*n* = 17), 7% were in Africa (*n* = 7), 6% were in the Near East (*n* = 6) and 2% in North America (*n* = 2).

### 3.1. Legume-Based Messaging in Dietary Guidelines

Most countries had at least one key message related to legumes (96.8%, *n* = 91); however, there was great variability in the language used and the detail provided (Table 1). In 33/91 countries, legumes were not specifically mentioned in the set of key messages; however, consumption was implied via a general message pertaining to the intake of all food groups, e.g., “Focus on meeting food group needs with nutrient-dense foods and beverages…” (United States). Legumes were mentioned as a unique dietary component in 20 dietary guidelines, e.g., “Eat dry beans, split peas, lentils and soya regularly” (South Africa), “Eat legumes like beans, lentils and green beans, daily” (Mexico) and “Eat less meat—choose legumes and fish” (Denmark), while 15 countries grouped legumes together with other protein-rich foods, e.g., “Eat some beans, pulses, fish, eggs, meat and other proteins”(The United Kingdom), “Lean meats and poultry, fish, eggs, tofu, nuts and seeds, and legumes/beans” (Australia). Countries in Africa more often highlighted legumes as a separate key message (*n* = 4/7), while countries in the APAC region more often group legumes together with other protein sources (*n* = 10/17).

Of the 91 countries with at least one legume-based key message, 42 utilised qualitative-based messaging and often used descriptors such as regularly, frequently or often to encourage consumption, e.g., “Consume legumes frequently” (Greece). Less than a quarter of countries outlined quantitative-based messages (*n* = 24) and highlighted specific serving frequencies, such as daily consumption, e.g., “Eat legumes daily” (Qatar) or encouraged intake at every meal, e.g., “Integrate grains and legumes, other grain and potatoes—in every main meal” (Albania).

### 3.2. Key Terms Used to Define Legumes

‘Legume’ was the most commonly used term to define the category in 42.6% of dietary guidelines (*n* = 40/94), followed by ‘Beans’ (13.8%, *n* = 13), ‘Pulses’ (10.6%, *n* = 10), ‘Peas and Beans’ (6.4%, *n* = 6), ‘Beans, Peas and Lentils (5.3%, *n* = 5) and ‘Pulses and Beans’ (2.1%, *n* = 2) (Table 1). Other terms used included ‘Beans, Lentils, Soybeans, Chickpeas’ (Slovenia), ‘Legume Seeds’ (Poland), ‘Legumes and Pulses’ (Thailand), ‘Soybeans’ (China), ‘Legumes/Beans’ (Australia) and a combination of ‘Legumes’ and ‘Peas, Kidney Beans, Lentils’, all of which were only used by one country each. Twelve countries did not have specific terminology, with no mention of legumes.

### 3.3. Recommended Serving Sizes and Frequency of Consumption

Overall, 39 countries provided a recommended serving frequency and 25 provided a recommended serving size for legumes (Table 1). Frequency of consumption ranged from two servings per day (Chile, Colombia and Paraguay) to two servings per week (Albania, France). In the United States, individuals should aim for 1–3 cups/week depending on energy needs and in Denmark the recommendation is to simply consume 100 g per day. The most common serving size was one-half a cup of cooked legumes (Kenya, Benin, Sierra Leone, Saudi Arabia, Oman, Australia (as a vegetable), Bangladesh and Austria), and ranged from 30 g fresh or cooked/10 g dried legumes (Estonia) to one cup of cooked legumes (Greece, Australia and New Zealand). Australia was the only country with two serving sizes for legumes: 75 g as a vegetable and 150 g as a meat alternative.

### 3.4. Food Group Classification

**Figure 1:**
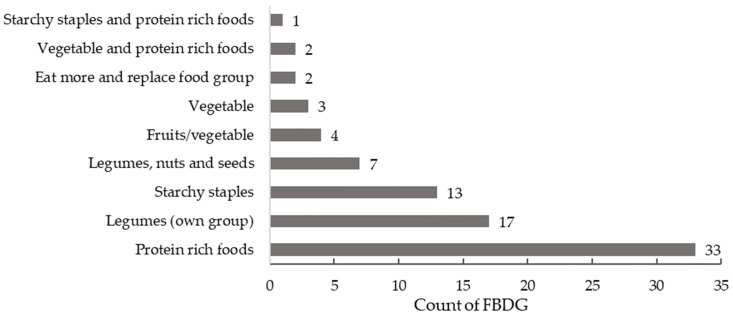
Classification of legumes by food group (*n* = 82). Excludes seven countries that did not have a food guide and five countries where the food group classification could not be determined (image of food guide was unclear).

Overall, 87/94 countries with FBDG had visual food guides. Legumes were mostly commonly included in the protein-rich food group (38%, *n* = 33), together with lean meats, poultry, fish, eggs, tofu and/or nuts and seeds. Legumes were a separate food group in 20% of countries (*n* = 17) and 13 countries grouped legumes together with other starchy staples (variously defined, however mostly included grains, cereals, tubers and/or cassava) (Figure 1). Seven FBDG grouped legumes with nuts and seeds (8%) and a smaller proportion included legumes in the fruits/ vegetable food group (5%, *n* = 4) and vegetable food group only (4%, *n* = 3). Overall, five FBDG grouped legumes into two foods groups. In Australia and the United States, legumes were considered both a vegetable and a meat alternative in the protein-rich food group. In Albania, legumes were classified in the protein-rich food group and the starchy staples group, while in Poland and Sweden, legumes featured in the “eat more” food group and the “replace/switch” food group (Table 1).

### 3.5. Legume Intake Data and Comparison to Legume Food Group Classification

**Figure A1:**
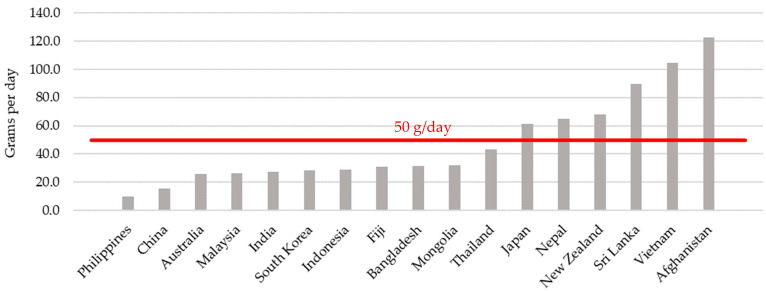
Median bean and legume intake for countries in the Asia and Pacific Region with food-based dietary guidelines (*n* = 17).

**Figure A2:**
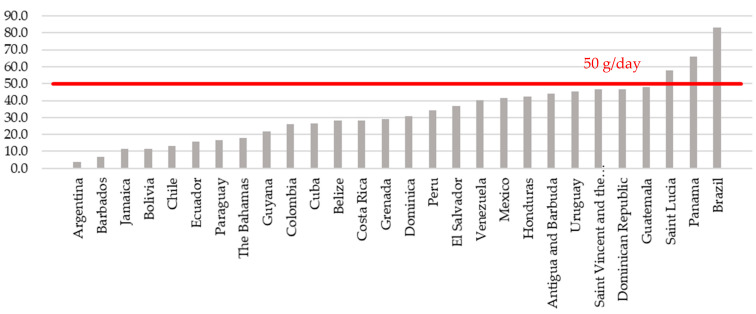
Median bean and legume intake for countries in Latin America and the Caribbean with food-based dietary guidelines (*n* = 28).

**Figure A3:**
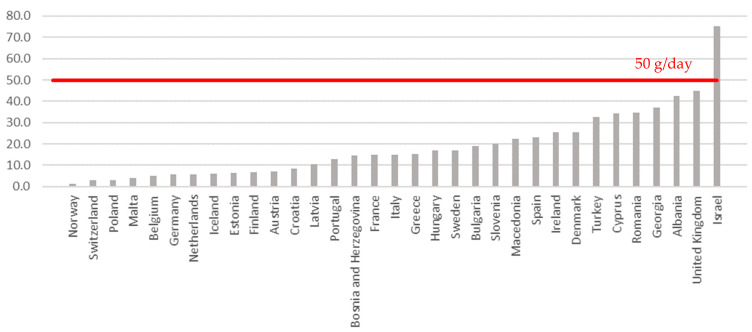
Median bean and legume intake for countries in Europe with food-based dietary guidelines (*n* = 33).

**Figure A4:**
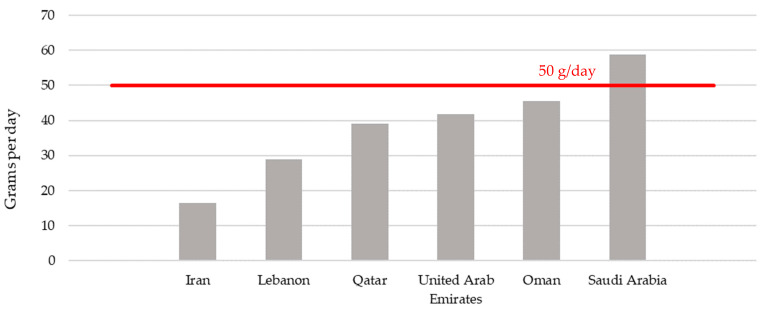
Median bean and legume intake for countries in the Near East with food-based dietary guidelines (*n* = 6).

**Figure A5:**
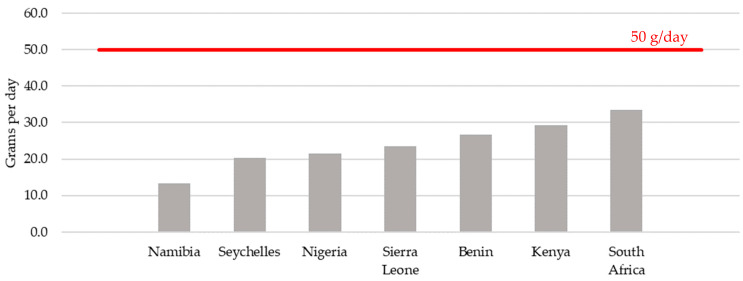
Median bean and legume intake for countries in Africa with food-based dietary guidelines (*n* = 7).

**Figure A6:**
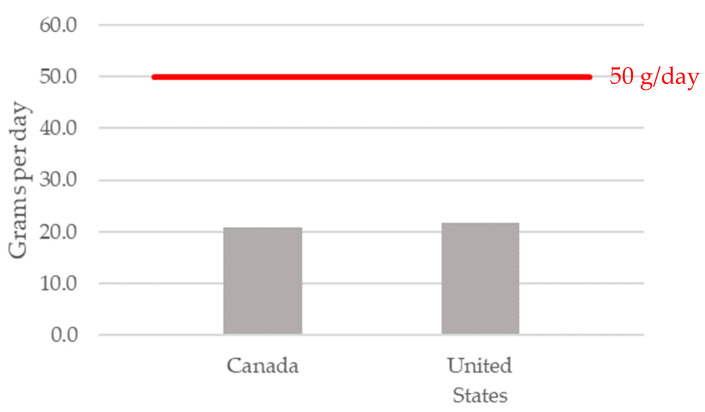
Median bean and legume intake for countries in North America with food-based dietary guidelines (*n* = 2).

Consumption data were available for 98.9% of countries with FBDG (*n* = 93/94) (Table 1) and of which, median intake ranged from 1.2 g/day (Norway) to 122.7 g/day (Afghanistan). At the regional level, the greatest range in intake for those with FBDG was seen in the APAC region (9.7–122.7 g/day) (Appendix A Figure A1), followed by LAC (3.7–83.0 g/day) (Appendix B Figure A2), Europe (1.2–75.3 g/day) (Appendix C Figure A3), Near East (16.4–58.9 g/day) (Appendix D Figure A4), Africa (13.4–33.6 g/day) (Appendix E Figure A5) and North America (20.8 to 21.8 g/day) (Appendix F Figure A6). Only 11 countries had a median intake >50 g/day, most of which were in the APAC region (*n* = 6/11).

All countries in the European region (*n* = 33) fell below 50 g of legumes per day, except Israel, and 36% (12/33) fell below 10 g/day indicating that Europe had the lowest level of reported consumption overall. In APAC, 65% of countries fell below 50 g/day (*n* = 11/17) but only two countries fell below 25 g/day. No country in Africa met the 50 g daily target, and 4/7 consumed below 25 g/day. More than two-thirds of countries in LAC (89%) fell below the consumption target of 50 g/day (*n* = 25/28), although most countries (*n* = 19/28) consumed above 25 g/day. Most countries in the Near East consumed above 25 g/day (*n* = 5/6), with just one country, Saudi Arabia, exceeding the 50 g/day target. Neither of the two countries representing North America met the 50 g target.

At the country-specific level, highest intakes were reported in Afghanistan (122.7 g/day), Vietnam (104.7 g/day), Sri Lanka (89.6 g/day), Brazil (83.0 g/day) and Israel (75.3 g/day), while Norway (1.2 g/day), Switzerland (2.9 g/day), Poland (3.0 g/day), Argentina (3.7 g/day) and Malta (4.0 g/day) reported the lowest consumption overall. In Australia and New Zealand, median legume intake was 26.1 and 68.0 g/day, respectively, and lower intakes were reported in Canada (20.8 g/day) and the United States (21.8 g/day). The UK reported a median intake of 44.8 g/day.

Median bean and legume consumption was compared to the legume food group classification for 81 countries with matched data. This excludes seven countries that did not have a food guide, five countries where the food group classification could not be determined and one country where consumption data was not available. In Albania, where legumes were classified as both a starchy staple and as a meat alternative in the protein-rich food group, median consumption was 42.4 g/day, higher than any other food group classification, although it should be noted that only one country grouped legumes as both a starchy staple and alternative to meat. Countries that placed legumes together with either nuts or seeds had the greatest range in intake (11.6–122.7 g/day), followed by those that grouped legumes together with protein-rich foods (4.0–104.7 g/day), which also had the greatest number of countries exceeding the 50 g/day target (5/33). More than two-thirds of those that classified legumes in their own food group (71%) consumed more than 25 g/day.

## 4. Discussion

Food-based dietary guidelines reflect a country’s particular pattern of eating while trying to optimise the health of the general population [30]. This global examination of dietary guidelines, focused on legumes, found that there was no consistency in the terminology used to define the category, with twelve different variations in the language used. While ‘legumes’, the more inclusive term, was utilised more frequently to define the category than the terms ‘beans’ or ‘pulses’ (referring to the dried, mature seeds), the discrepancy in the language used to categorise legumes may be a barrier to the dissemination of clear dietary guideline messaging, as previously mentioned by Didinger and Thompson (2021) [31]. Findings from this review, and previously published research [31,32], may provide impetus to revise the language and the classification system used to define legumes in dietary guidelines globally to move towards a more consistent, and accurate definition.

This review points to a particular opportunity for Australia to refer more specifically to ‘Beans, peas and lentils’, as opposed to ‘legumes/beans’ with the suggestion that the general terms, legumes and pulses, may not be familiar to consumers. This was reflected in a recent study involving 505 Australian respondents, in which legumes were most commonly associated with foods such as “beans” (72%), “lentils” (55%), “chickpeas” (42%), and “peas” (24%) [20]. Interestingly, a change in the terminology used to classify and define legumes has been adopted in the most recent revision of The United States 2020–2025 Dietary Guidelines, where the vegetable subgroup changed from ‘legumes (beans and peas)’ to ‘beans, peas and lentils’ [31,33]. A shift in the language used to define legumes in the ADG may assist in the translation of the guidelines and help consumers to understand what is expected in terms of consumption. However, it should be noted that consumer understanding and interpretation of dietary guidelines was not assessed in this review and further research is warranted.

Global bean and legume consumption drawn from the GDD 2018 was lower than suggested targets, with only 11 of those with FBDG reporting a median intake greater than 50 g/day. Although countries in the APAC region reported the highest level of consumption, this region also had the greatest variability (9.7–122.7 g/day), whereas surveys from North America had very little variation (20.8–21.8 g/day). Variability in consumption may be due to differences in cultural dietary patterns, where there may be a larger number of pulse consumers. For example, the highest level of consumption was reported in Afghanistan (122.7 g/day), where legumes are commonly incorporated into vegetarian and meat dishes as a thickener; however, this country was also one of few that presented the most explicit recommendations for the inclusion of legumes in the dietary pattern (Table 1). As a region, Europe, made up of 33 countries, had by far the lowest consumption of legumes, with more than one-third of countries (36%) reporting intakes less than 10 g/day.

In Australia, low consumption may be due to unfamiliarity with legumes and the tendency to use them as an ingredient rather than as a traditional pattern of eating or being central to the dish [34]. It has been suggested that increasing pulse consumption requires a multifaceted approach, with a focus on addressing the barriers to consumption, such as changing purchasing habits, or the provision of culinary education to improve knowledge and understanding on how to prepare and cook legumes [20]. The impact of COVID-19 on consumption of legumes is yet to be accounted for, with some indication that consumers may have pantry-stocked canned and dried legumes due to the uncertainty related to food supply issues. Future national nutrition surveys will need to consider methodological issues in detecting changes in consumption over the 2020–2021 time period, particularly considering food groups such as legumes. In any case, the history of poor intake of legumes in Australia may also be attributable, in part, to their lack of adequate representation within dietary guidelines. Findings from the present analysis provide some support for the need to address this in order to influence change and shift consumption.

Over time, traditional legume consumption may have been displaced through changes in dietary patterns and availability of alternate protein sources. A historical reflection from Germany in 1850 to 1970 reported a decrease in legume consumption and a rise in meat and fat intake, a trend that may be continuing today in some countries [35]. In Spain, over a shorter timeframe, legume consumption had decreased from 20.2 g/day (1991) to 11.9 g/day (2010) [36], and since then, intake has increased to 22.0 g/day following the recategorisation of legumes into the protein-rich food group. Changes in economic circumstances may also encourage a change from legumes to more animal proteins and the reverse may also be true. More recent attention on dietary sustainability issues, plant protein and flexitarian eating patterns may help draw attention to the relative proportion of animal and plant protein within dietary patterns. A historical review of legume recommendations in dietary guidelines with associated changes in consumption would be a suggested area for future research to determine the impact of dietary guidelines on intake.

Almost all food guides visually represented legumes, which were most often categorised as a meat alternative within the protein-rich food group (38%) or within their own group (20%). In contrast, when comparing placement of legumes in written guidelines with the relevant consumption data, higher consumption appeared aligned with placement of legumes in their own category or together with nuts and seeds (23 countries in total). This is closely followed by countries where legumes were recognised as a protein food (33 countries) and together with starchy staples. Australia and the US tended to have lower reported consumption, although legumes were classified in two groups which is based on their nutritional profile [11,19]; grouped together with vegetables despite being far higher in protein, and with meat and protein foods despite the high dietary fibre content. Including legumes in two separate food groups is purportedly to encourage consumption, although this approach was only utilised by these two countries. Furthermore, the phrase utilised in the Australian guidelines, “and/or legumes/beans” at the end of each food group statement could be considered tokenistic and may understate the importance of this food group in future dietary patterns.

While FBDG are built on the available scientific evidence supporting foods and dietary patterns, the statements within guidelines are designed to specifically suit the population. Similar to the terminology used to define the category, it was clear there was variation in the way guidelines were expressed based on culturally based dietary patterns. Countries such as Mexico, Qatar, Dominican Republic, Albania, Guatemala and Malta encouraged legume intake by categorising legumes as their own food group or provide detailed information on the culinary use of legumes to encourage consumption [30]. Other influences such as food security, historical effects, and the different structure of food chains in each country may also influence legume classification within FBDG [19].

This analysis represents the most comprehensive review of global FBDG and consumption data for legumes, however, should be viewed in light of the limitations. Full FBDG documents could not be reviewed for 49 countries, as they were not in English language which limits the scope the present study. The GDD 2018 dietary intake data are based on estimates using a combination of various sources, particularly self-reported dietary intake data. As such, there needs to be some consideration for the self-reported nature of dietary data collection, although this is true of all large population dietary data sets. In comparison to the GDD 2018, FAO estimates have shown to underestimate legume consumption by 50% [37], providing support for the use of the GDD. Furthermore, we acknowledge that legume content may be hidden as an ingredient in foods [38], particularly in countries where there is a more complex food supply system, this may perpetuate issues with underestimation of intake. We were unable to draw direct links between consumption data and the categorisation of legumes in dietary guidelines due to the variety of classification systems and low levels of consumption across numerous countries. However, there were some apparent patterns; particularly when legumes were grouped together with protein-rich foods or where legumes were classified in their own food group, consumption more often exceeded 25 g/day.

## 5. Conclusions

Dietary guidelines aim to facilitate easy translation to dietary patterns specific to a population. Legumes are potentially difficult to position due to their nutrient composition, being high in dietary fibre and plant-based protein. With greater emphasis on sustainable eating patterns, novel placement of legumes or at least repositioning of legumes may be a consideration within FBDG. Future research should test messaging in dietary guidelines, particularly in countries where increased consumption is desired and where legumes have been added to multiple food groups such as in Australia.

## Figures and Tables

**Table 1:** Legume-based messaging, food group classification, key legume terminology, recommended serving frequency, recommended serving size and median bean and legume consumption (in grams/day) for countries with FBDG (*n* = 94).

Country	Food Group Classification in Food Guide	Legume-Based Message/s	Key Legume Term	Recommended Serving Frequency ^a^	Recommended Serving Size	GDD Consumption (Median; 95% CI)
Africa						
Benin (2015)	Protein-rich foods	“When there is no meat, fish or eggs in a given day, you can replace them with pulses, peanuts, soybeans, soya, cheese or peas.”	Pulses	2–3 servings of food group/day *	½ cup (140 g) *	26.7 (22.7–31.5)
Kenya (2017)	Legumes, nuts and seeds (own group)	“Eat beans, peas, lentils, cowpeas, pigeon peas, soya, nuts and edible seeds regularly (at least four times a week).”	Beans, peas, lentils	4 serving/week	½ cup (125 mL) cooked dried beans, peas or lentils	29.2 (25.3–34)
Namibia (2000)	Protein-rich foods	“Eat beans or meat regularly”	Beans	n/s	n/s	13.4 (11.2–16.2)
Nigeria (2001)	Starchy staples	The diet should contain as wide a variety of foods as possible, e.g., legumes	Legumes	n/s	n/s	21.6 (18.7–24.8)
Seychelles (2006)	Fruits and/or vegetable	“Eat pulses (peas, beans and lentils) at least 4 times a week”	Pulses	4 servings/week	n/s	20.4 (17.1–24.4)
Sierra Leone (2016)	Legumes (own group)	“Eat beans, peas and lentils everyday”	Beans, peas and lentils	n/s	½ cup cooked beans/lentils	23.4 (20.0–27.7)
South Africa (2012)	Legumes (own group)	“Eat dry beans, split peas, lentils and soya regularly”	Beans, split peas, lentils and soya	n/s	n/s	33.6 (25.2–44.3)
Asia and the Pacific						
Afghanistan (2015)	Legumes, nuts and seeds (own group)	“Eat different types of food daily”	Pulses and beans	0.5–2 servings/day depending on energy needs.	½ cup (100 g) boiled lentil/peas, ¼ cup raw dry lentils/peas	122.7 (59.5–237.3)
Australia (2013)	Vegetable	“Plenty of vegetables, including different types and colours, and legumes/beans”	Legumes/beans	5 servings of the food group/day	½ cup (75 g) cooked dried or canned beans, peas or lentils	26.1 (18.7–36.5)
	Protein-rich foods	“Lean meats and poultry, fish, eggs, tofu, nuts and seeds, and legumes/beans”		2.5 servings of the food group/day	1 cup (150 g) cooked or canned legumes/beans	
Bangladesh (2013)	Protein-rich foods	“Consume required amounts of fish, meat, poultry, egg and legumes daily”	Legumes	1 serving/day. Combine cereals with legumes in a 3:1 ratio	⅓–½ cup pulses	31.7 (28.6–35.4)
China (2016)	Starchy staples	“Consume plenty of vegetables, milk, and soybeans”	Soybeans	250–400 g of the food group/day	n/s	15.8 (12.8–19.6)
Fiji (2018)	Protein-rich foods	“Eat body building foods such as dhal, dried peas and beans…”	Dhal, dried peas and beans	n/s	n/s	30.7 (17.9–53.9)
India (2011)	Starchy staples	“Eat variety of foods to ensure a balanced diet”	Pulses	2 servings/day for vegetarians, 1 serving/day for non-vegetarian	30 g	27.2 (24.6–30.2)
Indonesia (2014)	Protein-rich foods	“Eat high-protein foods (animal or vegetable source)”	n/s	2–4 servings of the food group/day	n/s	29.0 (25.5–33)
Japan (2010)	Cannot be determined **	“Combine vegetables, fruits, milk products, beans and fish in your diet.”	Beans	n/s	n/s	61.1 (53.5–70.5)
Malaysia (2010)	Protein-rich foods	“Consume moderate amounts of fish, meat, poultry, eggs, legumes and nuts.”	Legumes	½–1 serving/day	n/s	26.6 (22.6–31.1)
Mongolia (2010)	Cannot be determined **	“Consume a variety of nutrient-dense foods and beverages.”	n/s	n/s	n/s	32.0 (18.8–56)
Nepal (2012)	No food guide	“Eat pulses, fish, poultry, eggs and a little meat regularly.”	Pulses	n/s	n/s	64.7 (56.5–74.4)
New Zealand (2020)	Protein-rich foods	“Enjoy a variety of nutritious foods every day including: some legumes…”	Legumes	2 servings of the food group/day	1 cup (150 g) cooked or canned	68.0 (53.8–85.6)
Philippines (2012)	Protein-rich foods	“Consume fish, lean meat, poultry, eggs, dried beans or nuts daily for growth and repair of body tissues.”	Beans	3–4 servings of the food group/day	n/s	9.7 (8.6–11)
Republic of Korea (2016)	Protein-rich foods	“Eat a variety of foods including rice & other grains, vegetables, fruits, milk & dairy products, meat, fish, eggs, and beans.”	Beans	3–4 servings of the food group/day	n/s	28.5 (26.4–30.9)
Sri Lanka (2011)	Protein-rich foods	“Eat pulses, fish, dried fish, eggs, poultry and lean meat.”	Pulses	3–4 servings of the food group/day	3 Tbsp cooked pulses	89.6 (76.6–104.4)
Thailand (1998)	Protein-rich foods	“Eat fish, lean meat, eggs, legumes and pulses regularly.”	Legumes and pulses	n/s	n/s	43.4 (17.8–104.9)
Vietnam (2013)	Protein-rich foods	“Eat protein-rich foods from a good balance of vegetable and animal sources. Increase the intake of … beans/peas”	Beans/peas	n/s	n/s	104.7 (69.8–157.2)
Near East						
Iran (2015)	Legumes, nuts and seeds	“Eat legumes and dishes made with legumes once a day”	Legumes	n/s	n/s	16.4 (14.6–18.2)
Lebanon (2013)	Protein-rich foods	“Consume legume-based dishes regularly…”	Legumes	5–6.5 servings of the food group/day	¼ cup cooked legumes, 2 Tbsp hummus, 1 baked falafel	28.9 (24–34.7)
Oman (2009)	Legumes (own group)	“Consume one serving of legumes daily.”	Legumes	1 serving/day	½ cup cooked lentils, beans or peas, ¼ cup dried beans or tofu	45.5 (23.4–92.1)
Qatar (2015)	Legumes (own group)	“Eat legumes daily” “Choose legumes, nuts and seeds as alternative protein sources”	Legumes	n/s	n/s	39.0 (19.5–85.9)
Saudi Arabia (2013)	Protein-rich foods	“Enjoy a variety of food items from major food groups daily—Include legumes, nuts, seeds, poultry, and lean meats in your eating pattern”	Legumes	2–3 servings of the food group/day	½ cup cooked legumes	58.9 (31.1–110.7)
United Arab Emirates (2019)	Cannot be determined **	“Consume diversified nutrient-rich foods and beverages.”	n/s	n/s	n/s	41.7 (21.5–84.2)
Europe						
Albania (2008)	Starchy staples	“Integral grains and legumes, other grain and potatoes—in every main meal”	Legumes	2 servings/ day	60–100 g legumes	42.4 (33.3–54)
	Protein-rich foods	“Substitute greasy meat and meat by-products with peas, kidney beans, lentils…”	Peas, kidney beans, lentils			
Austria (2015)	Vegetables, legumes and fruits	“Eat five servings of vegetables, legumes and fruits every day. The ideal would be to eat three servings of vegetables and/or legumes…”	Legumes	3 servings of vegetables and/or legumes/day *	70–100 g dry pulses (150–200 g cooked) *	7.0 (4.6–10.8)
Belgium (French region) (2020)	Protein-rich foods	n/s	Legumes	n/s	n/s	5.0 (3.4–7.4)
Bosnia and Herzegovina (2004)	Cannot be determined **	“Eat meat, poultry, eggs and legumes several times a week”	Legumes	n/s	n/s	14.6 (8.7–24.8)
Bulgaria (2006)	Protein-rich foods	“Replace meat and meat products often with fish, poultry or pulses”	Pulses	2 servings per week	200–300 g	18.9 (13.7–25.9)
Croatia (2002)	Starchy staples	n/s	n/s	n/s	n/s	8.6 (3.4–21.8)
Cyprus (2007)	Legumes (own group)	“Consume a traditional Mediterranean diet with lots of legumes…” “Increase your intake of fibre and complex carbohydrates by using whole grains, legumes, vegetables and fruit with the peel on”	Legumes	n/s	n/s	34.3 (13.3–88.3)
Denmark (2021)	Legumes (own group)	“Eat less meat—choose legumes and fish”	Legumes	100 g/day	n/s	25.5 (15.6–42.2)
Estonia (2017)	Vegetable	“Increase the consumption of vegetables, including legumes.”	Legumes	3–4 servings/ week *	30 g fresh or cooked, 10 g dried legumes *	6.3 (4.9–8.2)
Finland (2014)	Vegetable, fruits and berries	“Eat vegetables, fruits and berries frequently…”	n/s	500 g of the food group/day	n/s	6.7 (5.4–8.5)
France (2019)	No guide	“The consumption of pulses (beans, lentils, chickpeas, etc), at least twice a week”	Pulses	2 servings/week	n/s	14.9 (12.7–17.5)
Georgia (2005)	Protein-rich foods	“Replace fatty meat and meat products with legumes…”	Legumes	1–3 servings/d (150–200 g)	¼ cup of beans	36.9 (21.5–65.3)
Germany (2017)	Vegetable	“Enjoy a variety of foods”.	n/s	n/s	n/s	5.7 (5.1–6.5)
Greece (2014)	Legumes, nuts and seeds	“Consume legumes frequently”	Legumes	3 servings/week *	1 cup of cooked drained legumes (150–200 g) *	15.2 (11.5–19.9)
Hungary (2004)	Fruits and vegetables	“Eat dark green vegetables, citrus fruits, tomato and legumes often..”	Legumes	n/s	n/s	16.9 (5.4–51)
Iceland (2014)	Protein-rich foods	“Variety of foods in reasonable quantity”	Beans	2 servings of food group/day *	¾ cup beans or lentils *	6.0 (4.7–7.7)
Ireland (2015–16)	Protein-rich foods	“Choose eggs, beans and nuts”	Beans	n/s	n/s	25.5 (11–56.4)
Israel (2008)	Protein-rich foods	“Choose fibre-containing foods such as… legumes…”	Legumes	n/s	n/s	75.3 (64–88.5)
Italy (2019)	No guide	“Eat whole grain and legumes”	Legumes	n/s	n/s	14.9 (13.2–16.9)
Latvia (2008)	Protein-rich foods	“Eat legumes, fish or lean meat. The recommended daily amount of those products is 2–3 servings”	Legumes	2–3 servings of food group/day	n/s	10.5 (4.9–23.5)
Malta (2016)	Protein-rich foods	“Include legume-based dishes throughout the week. These could take the form of home-made dips (bigilla, red kidney dip and hummus). Salads (bean and chickpea salad), stews, vegetable soups (minestra) and home-made torta talful.”	Legumes	2+ servings/week	70 g (raw), 140 g (cooked/canned)	4.0 (2.1–7.5)
The Netherlands (2016)	Protein-rich foods	“Eat less meat and more plant-based foods, and vary with fish, pulses, nuts, eggs and vegetarian products”	Pulses	n/s	n/s	5.9 (5.0–6.9)
The Republic of North Macedonia (2014)	No guide	“Substitute meat and meat products with fish, poultry, beans and bean-based products”	Beans	n/s	n/s	22.4 (13.6–38.2)
Norway (2014)	No guide	n/s	n/s	n/s	n/s	1.2 (0.5–3.1)
Poland (2020)	Eat more food group	“Eat more legume seeds (e.g., beans, peas, chickpeas, lentils, broad beans)”	Legume seeds	n/s	n/s	3.0 (2.6–3.4)
	Replace food group	“Replace red meat and processed meat with fish, poultry, eggs, legume seeds and nuts”		n/s	n/s	
Portugal (2003)	Legumes (own group)	“Eat foods from each food group every day to have a complete diet.”	Legumes	1–2 servings/day	1 Tbsp raw dried (25 g), 3 Tbsp raw fresh (80 g), 3 Tbsp dried/cooked fresh (80 g)	12.8 (11.3–14.4)
Romania (2006)	Cannot be determined **	“Eat a variety of foods”	n/s	n/s	n/s	34.6 (27.8–43.5)
Slovenia (2011)	Protein-rich foods	“Eat a variety of foods originating mainly from plants, rather than animals”.	Beans, lentils, soybeans, chickpeas.	3–5 servings of the food groups/day *	4 tablespoons of beans, lentils, soybeans, chickpeas *	20.1 (8.9–44.4)
Spain (2008)	Protein-rich foods	“Enjoy a variety of foods”	Legumes	n/s	n/s	22.9 (10.7–49.5)
Sweden (2015)	“Eat More”	“Eat more vegetables and fruit—Ideally, choose high fibre vegs such as… beans.”	Beans	n/s	n/s	17.0 (14.7–19.6)
	Switch To	“Soups, pies and stir fries can easily be made without meat”		n/s	n/s	
Switzerland (2011)	Starchy staples	“Consume three portions of grains, potatoes and pulses per day”	Pulses	3 servings of the food group/day	n/s	2.9 (2.1–4.1)
Turkey (2014)	Protein-rich foods	“Increase consumption of wholegrain cereals and leguminous seeds”	Legumes	2 servings of food group/day	90 g	32.6 (21.9–49.3)
United Kingdom (2016)	Protein-rich foods	“Eat some beans, pulses, fish, eggs, meat and other proteins..”	Beans and pulses	n/s	80 g	44.8 (36.8–55.2)
Latin America and the Caribbean						
Antigua and Barbuda (2013)	Legumes, nuts and seeds	“Choose to eat a variety of foods every day”	Peas and beans	n/s	n/s	44.2 (26.2–75.7)
Argentina (2014)	Starchy staples	“Eat legumes; cereals, preferably wholemeal; potato; sweet potato; corn or cassava”	Legumes	n/s	n/s	3.7 (2.7–4.9)
Bahamas (2002)	Legumes (own group)	“Make starchy vegetables, peas and beans a part of your diet”	Peas and beans	n/s	n/s	18 (10.1–32.4)
Barbados (2017)	Legumes (own group)	“Enjoy a wide variety of foods every day”	Legumes	n/s	n/s	6.9 (4.4–11)
Belize (2012)	Legumes (own group)	“Choose different types of foods from all the food groups daily”	Legumes	1–2 servings/day depending on energy needs	¼ cup red beans or lentils	28.2 (16.4–49.7)
Bolivia (2013)	Starchy staples	“Consume a varied diet daily, including foods from all groups”	n/s	n/s	n/s	11.7 (10–13.6)
Brazil (2014)	No guide	“Make natural or minimally processed foods part of your diet”	Beans	n/s	n/s	83.0 (71.6–96.2)
Chile (2013)	Protein-rich foods	“To keep your heart healthy…eat legumes at least twice a week, without mixing them with cold or cured meats”	Legumes	2 servings/ week	n/s	13.1 (7.4–23.2)
Colombia (2015)	Protein-rich foods	“To complement your diet, eat pulses like beans, lentils, peas and chickpeas at least two times per week”	Pulses	2 servings/ week	n/s	26.1 (22.8–29.9)
Costa Rica (2010)	Starchy staples	“Eat rice and beans; they are the basis of the everyday diet”	Beans	n/s	n/s	28.4 (16.9–49.7)
Cuba (2009)	Protein-rich foods	“A variety of foods during the day is pleasant and necessary for good health”	Legumes	n/s	n/s	26.4 (16–44.8)
Dominica (2007)	Legumes (own group)	“Always try to eat a variety of foods every day. Use the basket to help you make the right choices”	Peas and Beans	n/s	n/s	30.9 (18.6–53.5)
Dominican Republic (2009)	Legumes (own group)	“Increase consumption of beans, grains, fish, eggs and dairy to keep your bones and organs healthy”	Beans	n/s	n/s	46.8 (40.9–54.1)
Ecuador (2018)	Protein-rich foods	“Let’s include animal source foods or legumes in our daily dishes to develop and strengthen our bodies” “Let’s eat better by combining legumes with cereals like rice, maize or quinoa”	Legumes	n/s	n/s	15.8 (8.9–28)
El Salvador (2012)	No guide	“Prepare varied meals using natural foods every day”	Beans	n/s	n/s	36.8 (21.8–63.8)
Grenada (2006)	Legumes (own group)	“Eat a variety of foods”	Legumes	n/s	n/s	29.1 (17.7–50.2)
Guatemala (2012)	Starchy Staples	“Eat beans and tortillas every day: eat two tablespoons of beans per tortilla, because they provide more nutrients and fill you up more.”	Beans	n/s	n/s	48.1 (41.7–55.5)
Guyana (2018)	Legumes (own group)	“Eat different types of foods from all the food groups daily”	Legumes	n/s	n/s	21.9 (17.6–27)
Honduras (2013)	Starchy staples	“Eat foods from all food groups to enjoy good health”	n/s	n/s	n/s	42.4 (36.7–49.1)
Jamaica (2015)	Legumes, nuts and seeds	“Include peas, beans and nuts in your daily meals”	Legumes	3 servings of food group/day	¼ cup cooked peas, beans, baked beans, stewed peas and chickpeas (1 Tbspn), ½ cup canned green peas and lentils	11.6 (7.9–16.9)
Mexico (2015)	Legumes (own group)	“Include the three food groups: fruits and vegetables, legumes and animal source foods in your breakfast, lunch and dinner” “Eat legumes like beans, lentils and green beans, daily”	Legumes	n/s	n/s	41.4 (37.3–45.9)
Panama (2013)	Starchy staples	“Eat a variety of foods every day”	n/s	n/s	n/s	66.1 (40.8–109.8)
Paraguay (2015)	Protein-rich foods	“To have a healthy diet, eat from all 7 food groups (… meats, legumes and eggs…) every day” “Eat cereals and legumes 2 times a week because together they are more nutritious”	Legumes	2 servings/week consumed with cereals	n/s	16.7 (10.2–28.9)
Peru (2019)	Starchy staples	“Don’t miss legumes, they are tasty, healthy and can be prepared in many ways”	Legumes	n/s	n/s	34.4 (30.3–39.5)
Saint Kitts and Nevis (2010)	Legumes, nuts and seeds (own group)	“Every day, choose foods from each of the groups (shown on the mill)”	Peas and beans	n/s	n/s	No data
Saint Lucia (2007)	Legumes (own group)	“Always try to eat ground provisions, peas and beans in your meals every day”	Peas and beans	n/s	n/s	57.6 (34.2–98.3)
Saint Vincent and the Grenadines (2006)	Legumes (own group)	“Eat a variety of foods from the foods groups in the breadfruit”	Legumes	n/s	n/s	46.6 (28.2–79.2)
Uruguay (2016)	Vegetable	“Incorporate vegetables and fruits in all your meals. This will help you to feel good and to maintain a healthy weight”	Legumes	n/s	n/s	45.6 (26.4–77.6)
Venezuela (1991)	Starchy staples	“Eat a varied diet. Get the fibre that your body needs from plant foods on a daily basis”	n/s	n/s	n/s	40.4 (23.9–71.7)
North America						
Canada (2019)	Protein-rich foods	“Eat plenty of vegetables and fruits, whole grain foods and protein foods. Choose protein foods that come from plants more often”	Legumes	n/s	n/s	20.8 (18–24.2)
United States (2020)	Vegetable	“Focus on meeting food group needs with nutrient-dense foods and beverages…”	Beans, Peas, Lentils	1–3 cups/ week depending on energy needs	n/s	21.8 (20.2–23.6)
	Protein-rich foods				n/s	

* Translated; ** Food guide image unclear; ^a^ Recommended serving frequency relates to legumes specifically unless stated otherwise. n/s = not specific.

## Data Availability

All data on food-based dietary guidelines are contained within the article. Publicly available datasets on bean and legume consumption were analysed and presented as Figure A1, Figure A2, Figure A3, Figure A4, Figure A5 and Figure A6. Consumption data can be found via the Global Dietary Database: https://www.globaldietarydatabase.org/ (accessed on 15 June 2022).
